# Exploring the determinants of health literacy and its effects on health-related outcomes in family caregivers of patients with cancer

**DOI:** 10.1007/s00520-025-09494-7

**Published:** 2025-04-29

**Authors:** Sumin Park, Eric C. Blackstone, Susan R. Mazanec

**Affiliations:** 1 College of Nursing, University of Utah, Salt Lake City, UT USA; 2 Division of Population Sciences, Dana-Farber Cancer Institute, Boston, MA USA; 3 Frances Payne Bolton School of Nursing, Case Western Reserve University, Cleveland, OH USA

**Keywords:** Cancer, Caregivers, Health literacy, Radiation treatment, Caregiver-reported health outcomes, Structural equation modeling

## Abstract

**Purpose:**

The health literacy of family caregivers (CGs) of patients with cancer is an essential factor that needs to be addressed given their vital roles in cancer care. Yet, little is known about the factors associated with the health literacy of cancer CGs, particularly during the initial stage of cancer treatment. We aimed to examine the factors associated with health literacy in CGs of patients with cancer and their impact on health-related outcomes prior to the beginning of radiation treatment.

**Methods:**

Baseline data from an ongoing randomized controlled trial were used. The sample included CGs of patients with cancer, the majority of whom were white, non-Hispanic/Latino, spouses/partners, with incomes above $50,000 and college degrees or higher. Structural equation modeling was conducted using maximum likelihood estimation to examine the paths between demographic variables, CG health literacy, and health-related outcomes.

**Results:**

Out of 236 CGs, one-third had limited to marginal health literacy. CGs with higher annual household income (β = .20, *p* = .009) were more likely to have higher levels of health literacy, which was associated with better overall health (β = .23, *p* = .033), lower anxiety (β = -.25, *p* = .001), and reduced fatigue (β = -.25, *p* = .011).

**Conclusion:**

CGs with lower health literacy face greater risks of poor health, anxiety, and fatigue, which could impact their caregiving ability. Targeted interventions and practical strategies (e.g., simplified education materials, teach-back methods, health literacy screening) are needed to support CGs with limited health literacy, ensuring they have adequate resources to navigate complex cancer care. Future longitudinal studies should explore these relationships to understand long-term impacts.

**Trial registration number:**

NCT04055948

**Date of registration:**

08/14/2019

## Introduction

According to Healthy People 2030, personal health literacy (hereinafter called health literacy), one of the social determinants of health, is defined as the extent to which individuals can find, comprehend, and utilize information and services to make informed health-related decisions and actions [1]. Likewise, the World Health Organization emphasizes that health literacy is shaped by organizational structures and accessible resources [2]. Low health literacy is a social risk that can impact an individual’s treatment and health service use, health behaviors (e.g., physical activity, medication adherence), health-related outcomes (e.g., quality of life), and mortality [3–5]. Despite the crucial importance of health literacy, only 12% of the US population has a proficient level, indicating that 88% lack the necessary skills to understand and effectively utilize lengthy, complex, and abstract health information, according to the National Assessment of Adult Literacy survey [6].

The health literacy of family caregivers (CGs) of patients with cancer is an essential factor that needs to be addressed, given their vital roles throughout patients’ cancer care trajectories. Such roles include obtaining, assessing, and interpreting information related to cancer diagnosis, staging, treatment, and survivorship [7]. Family CGs also engage in various medical and nursing-related tasks, such as helping patients take medications, monitoring symptoms and treatment side effects, and/or managing tubes or drainages [8–10]. In addition, CGs communicate with healthcare providers and participate in the decision-making process for patient care [8, 9]. Family CGs actively interact with healthcare providers seeking illness- and treatment-related information with and/or on behalf of the patients [8, 9, 11]. Low health literacy in CGs may impede their ability to recognize patients’ needs, communicate effectively with both patients and healthcare providers, and relay information to other family members [7, 12]. Particularly during the initial phase of cancer treatment, a substantial amount of information is provided within a short timeframe, introducing complex treatment options (i.e., surgery, chemotherapy, radiation treatment, immunotherapy) tailored to the diagnosis and progression of the disease [10, 13]. Prior to the beginning of radiation treatment, consultation involves detailed discussions on the purpose and rationale of the treatment, potential side effects, and the logistics of radiation treatment. These discussions are further complicated by the highly technical nature of radiation oncology, making it challenging for family caregivers to fully comprehend the information [14]. Consequently, the low health literacy of CGs may exacerbate the challenges and stress associated with this phase and lead to suboptimal treatment decision-making.

Previous studies have found that CG’s health literacy is associated with cancer survivors’ psychological outcomes, including depression and health-related quality of life [15] and CG’s distress, anxiety, burden, and self-efficacy [16, 17]. Yet, there remains limited knowledge about the factors influencing the health literacy of cancer patients'CGs. Additionally, the impact of CGs’ health literacy on their own health outcomes is not well understood, particularly during the initial stages of cancer treatment, when complex treatment options are introduced.

This study was guided by the Health Literacy Skills Conceptual Framework [18]. The conceptual framework postulates the relationships between background factors (e.g., demographic characteristics, resources, prior knowledge), development and use of health literacy, mediating factors, and health-related outcomes. The conceptual framework posits that individuals’ demographic characteristics (e.g., age, race), prior knowledge (e.g., illness experience, healthcare knowledge), resources (e.g., socioeconomic status, education, language, social support), and capabilities (e.g., vision, hearing) influence health literacy. In turn, health literacy affects health-related behaviors and outcomes (e.g., health/well-being, quality of life, morbidity/mortality), with these relationships potentially mediated by factors such as attitudes, motivation, and self-efficacy.

The purpose of this study was to examine the factors associated with health literacy in CGs of patients with cancer prior to the beginning of radiation treatment. The specific aims were to (1) measure health literacy in CGs of patients with cancer prior to the beginning of radiation treatment, and (2) examine the relationship between CG demographic variables–age, gender, race, socioeconomic status (measured by annual household income), education level, previous caregiving experience, previous experience working as a professional caregiver– health literacy, and health-related outcomes (anxiety, fatigue, physical health, mental health). We hypothesized that CG resources (education level, socioeconomic status) and prior knowledge (previous caregiving experience, previous experience working as a professional caregiver) influence CG health literacy, and that CG health literacy influences CG health-related outcomes (anxiety, fatigue, physical health, mental health).

## Methods

### Setting and sample

A descriptive, correlational, cross-sectional study design was used. The study utilized baseline data (pre-randomization and before starting radiation treatment) from an ongoing randomized controlled trial – *Building Family Caregiver Skills Using a Simulation-Based Intervention for Care of Patients with Cancer* (R37 CA240707; Mazanec, PI). The parent study is being conducted at multiple sites in Northeast Ohio.

The aim of the parent study is to test an intervention in which a nurse interventionist meets with CGs to provide support, information, and skills training using simulation. A convenience sampling method is used to recruit subjects. Participant eligibility was pre-screened using the patient’s medical chart. Eligible patients were approached during their radiation treatment consultation and simulation. Once both the patient and caregiver provided consent to participate, baseline data were collected before the start of radiation treatment. This manuscript includes baseline data collected between January 1, 2020, and May 30, 2024. To ensure a diverse caregiver population, recruitment was conducted across multiple sites, including a safety-net hospital.

Patients are eligible if they are: (a) over the age of 18, (b) diagnosed with stage I – IV A/B head and neck cancer; stage I – IVA esophageal cancer; stage I – III anal/rectal cancer, and stage II – III non-small cell lung cancer, (c) receiving radiation treatment, and (d) has a CG involved in his/her care. Family CGs are eligible if they are (a) over the age of 18 and (b) identified as the primary CG for the patient. Exclusion criteria for patients include those (a) without a CG or (b) receiving hospice care. Exclusion criteria for CGs include those who are currently undergoing active cancer treatment themselves because actively treating their own cancer could significantly influence their physical and mental health, potentially confounding the results. The parent study is approved by the Institutional Review Board of University Hospitals Cleveland Medical Center (Study 20,190,943) and written informed consent is obtained from the study participants. A sample size of 223 is sufficient to detect an effect size of 0.20 with 2 latent variables and 7 observed variables at a significance level of 0.05 and a power of 0.80 [19].

### Measures

#### Demographic variables

Demographic characteristics are self-reported by CGs. These demographic characteristics include age, gender, race/ethnicity, education, socioeconomic status (annual household income), previous experience working as a professional caregiver (e.g., doctor, nurse, or social worker), and previous caregiving experience.

#### Health literacy

The Brief Health Literacy Screening Tool (BRIEF) was used to measure CG health literacy [20]. The scale consists of 4 items on a 5-point Likert scale. Total scores are the sum of four items ranging from 4 to 20, with higher scores reflecting higher health literacy. The BRIEF scores are categorized as limited (4–12), marginal (13–16), and adequate (17–20). The Cronbach’s alpha for this study was 0.73. The measure has demonstrated good psychometric properties, including concurrent validity, and has been utilized in CG populations, including those caring for cancer patients [20, 21].

#### Anxiety

The Patient-Reported Outcomes Measurement Information System (PROMIS®) Short Form v1.0-Anxiety 7a was used to measure CG anxiety [22]. The scale consists of 7 items on a 5-point Likert scale ranging from 1 (never) to 5 (always). The total summed raw scores (ranging from 7 to 35) were converted to t-scores (ranging from 36.3 to 82.7). The higher t-core indicates a higher anxiety level. The Cronbach’s alpha in this study was 0.92, and the measure has demonstrated good psychometric properties including concurrent validity in cancer CG [22–24].

#### Fatigue

The PROMIS® Short Form v1.0-Fatigue 7a was used to measure CG fatigue [25]. The scale has 7 items on a 5-point Likert scale ranging from 1 (never) to 5 (always). The total summed raw scores (ranging from 7 to 35) were converted to t-scores (ranging from 29.4 to 83.2). The higher t-score indicates a greater fatigue level. The Cronbach’s alpha in this study was 0.84, and the measure has well-established psychometric properties in the cancer CG population [24, 25].

#### Physical and mental health

The PROMIS® Form v1.2-Global Health was used to measure CG physical and mental health [26]. Each physical and mental health consists of 4 items. Total summed raw scores for physical health (ranging from 4 to 20) were converted to t-scores ranging from 16.2 to 67.7, while those for mental health (also ranging from 4 to 20) were converted to t-scores ranging from 21.2 to 67.6. The Cronbach’s alpha ranged from 0.70 to 0.81 in this study, and the measure demonstrated excellent psychometric properties, including among cancer CGs [27, 28]

### Statistical analysis

Descriptive statistics, including mean, SD, range, number, and percentages, were conducted to identify the participants’ demographic characteristics and study variables using IBM Statistical Package for Social Sciences version 29. T-tests, Pearson’s correlation, and Spearman’s rank correlation were computed to examine the preliminary relationships between demographic variables (age, gender, race, socioeconomic status, education level, previous caregiving experience, previous experience working as a professional caregiver), health literacy, and health-related outcomes (anxiety, fatigue, physical health, mental health). Structural equation modeling was conducted using maximum likelihood estimation, which also managed missing data through maximum likelihood to evaluate the model fit (AMOS version 29). The significant factors identified in correlation analyses were incorporated into the structural equation model. The model fit was assessed using the Tucker-Lewis index (TLI), comparative fit index (CFI), and root mean square error of approximation (RMSEA). A model with a TLI > 0.90, CFI > 0.90, and RMSEA < 0.08 were considered to have an acceptable fit [29]. A statistical significance level of 0.05 was set for the estimated paths, and post hoc modifications were conducted to incorporate additional paths.

## Results

### Participant characteristics

Baseline data as of May 30, 2024, which consisted of 236 CGs, were included in the analysis. Demographic characteristics are presented in Table 1. The mean age of CGs was 58.46, ranging from 19 to 89. Most were female (78.3%), white (81.6%), non-Hispanic/Latino (99.0%), and spouse or partner of the patients (65.2%). More than half of the CGs had a college degree or higher and an annual household income exceeding $50,000. Approximately 65% of CGs had previous experience caring for family members or friends, and 20.5% had previous experience working as a professional caregiver, including roles such as doctor, nurse, or social worker.

**Table 1:** Family Caregiver Demographic Characteristics (N= 236)

Variables	n (%)	M (SD)
Age		58.46 (13.46) Range: 19–89
Gender		
Female	184 (78.3)	
Male	51 (21.7)	
Race		
Black or African American	39 (16.5)	
Multi-race	4 (1.7)	
White	191 (81.6)	
Ethnicity		
Non-Hispanic/Latino	192 (99.0)	
Hispanic/Latino	2 (1.0)	
Relationship to Patient		
Spouse/Partner	154 (65.2)	
Sibling	18 (7.6)	
Parent	10 (4.2)	
Child	36 (15.3)	
Friend	7 (3.0)	
Other	11 (4.7)	
Marital Status		
Single	32 (13.6)	
Married/Partnered	178 (75.4)	
Separated/Divorced/Widowed	26 (11.0)	
Employment Status		
Employed (part or full-time)	121 (51.2)	
Retired	80 (33.9)	
Disabled/Not Employed	23 (9.8)	
Other	12 (5.1)	
Education		
Less than Highschool	2 (0.9)	
Highschool Diploma/GED	94 (40.0)	
College	105 (44.7)	
Post-Graduate	34 (14.5)	
Annual Household Income		
$20,000 or less	25 (11.2)	
$20,001 to $49,999	51 (22.9)	
$50,000 to $99,999	80 (35.9)	
$100,000 or greater	67 (30.0)	
Previous Caregiving Experience		
Yes	152 (64.7)	
No	83 (35.3)	
Previous Experience as a Professional Caregiver (e.g., doctor, nurse, social worker)		
Yes	48 (20.5)	
No	186 (79.5)	

### Study variables

Table 2 presents the study variable scores. The mean health literacy score was 17.27 (SD = 2.75), placing it on the higher end of the possible range. However, among 236 CGs, 17 (7.2%) and 60 (25.5%) had limited and marginal health literacy scores, respectively. CG's physical and mental health, as well as fatigue, had mean scores close to the U.S. population norm of 50. However, the mean CG anxiety score prior to the start of patient radiation treatment was 53.48 (SD = 7.91), exceeding the U.S. population norm of 50.

**Table 2:** Study variables (N= 236)

	M (SD)	n(%)	95% CI	Possible range
Health Literacy	17.27 (2.75)		16.91–17.64	4–20
Limited		17 (7.2)		
Marginal		60 (25.5)		
Adequate		158 (67.2)		
Mental Health^a^	50.31 (7.79)		49.28–51.35	21.2–67.6
Physical Health^a^	51.46 (7.29)		50.49–52.43	16.2–67.7
Anxiety^a^	53.48 (7.91)		52.43–54.54	36.3–82.7
Fatigue^a^	49.29 (7.16)		48.34–50.24	29.4–83.2

^a^T-scores

### Associations among study variables

Table 3 presents the correlations among study variables. Pearson’s correlation and Spearman’s rank correlation analysis results showed that high health literacy was associated with higher education (*ρ* = 0.22, *p* < 0.001) and higher annual household income (*ρ* = 0.23, *p* = 0.002). Also, high health literacy was related to better mental health (*r* = 0.22, *p* < 0.001), lower anxiety (*r* = − 0.20, *p* = 0.002), and lower fatigue (*r* = − 0.20, *p* = 0.002). CGs with previous caregiving experience reported higher health literacy; *t* (232) = − 1.97, *p* = 0.049. There was no significant relationship between health literacy and age, gender, race/ethnicity, or previous experience working as a professional caregiver.

**Table 3:** Correlations among study variables

	1	2	3	4	5	6	7	8
1. Health literacy	-							
2. Age	.01	-						
3. Education	.22***	-.01	-					
4. Annual household income	.23***	.00		-				
5. Physical health	.12	.09	.04	.18**	-			
6. Mental health	.22***	.21**	.07	.23***	.62***	-		
7. Anxiety	-.20**	-.18**	.02	-.11	-.35***	-.57***	-	
8. Fatigue	-.20**	-.16*	.00	-.18**	-.66***	-.58***	.49***	-

*p<.05, **p<.01, ***p <.001

### Model test

**Fig. 1:**
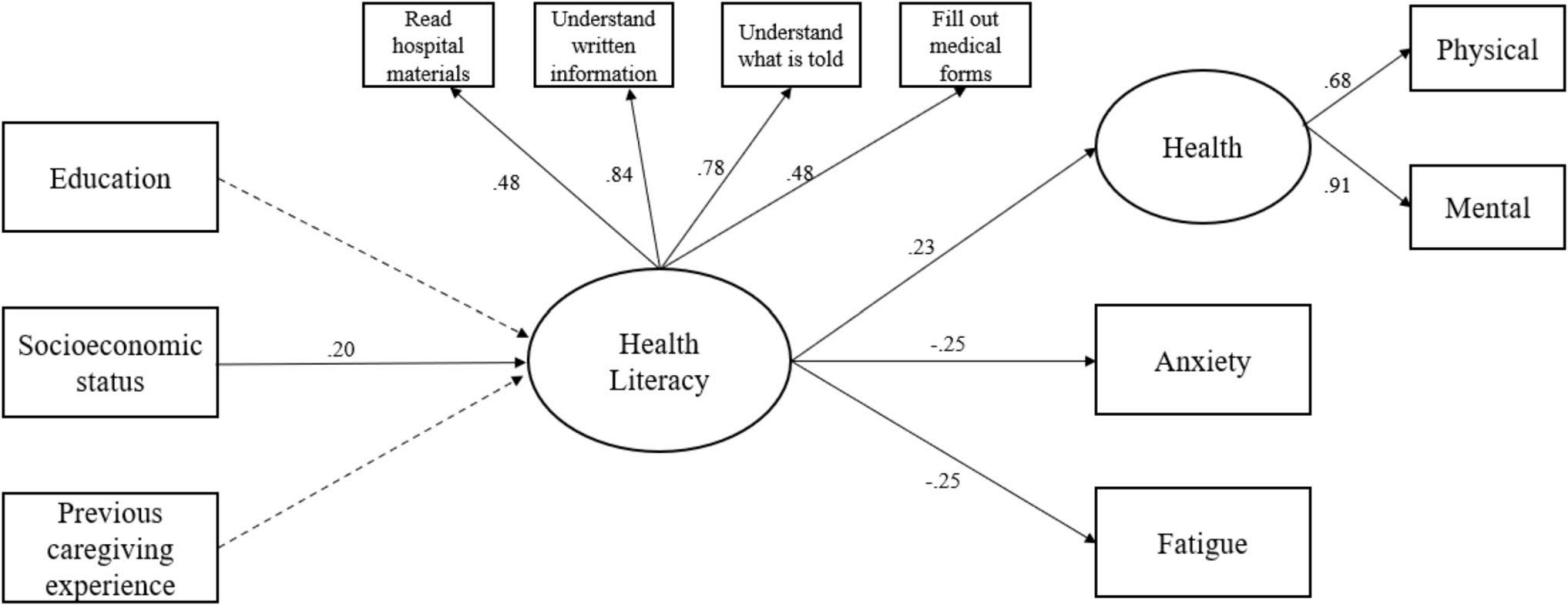
Structural equation model with standardized loadings. ^a^dotted lines indicate an insignificant path and solid lines indicate a significant path. ^b^socioeconomic status was measured by annual household income

Based on the correlation analysis results, insignificant path from the initial hypothesized model (previous experience working as a professional caregiver health literacy), was removed in the structural equation model to improve model fit. The final structural equation model demonstrated an acceptable fit to the data, as indicated by the following fit indices: TLI = 0.92, CFI = 0.95, and RMSEA = 0.06. Standardized path coefficients for the final model are presented in Fig. 1. Family CGs’ annual household income positively predicted health literacy, showing a small effect (*β* = 0.20, *p* = 0.009). However, other demographic variables (education, previous caregiving experience, or previous experience working as a professional caregiver) were not related to health literacy. Family CGs’ health literacy significantly predicted health (*β* = 0.23, *p* = 0.033), anxiety (*β* = − 0.25, *p* = 0.001), and fatigue (*β* = − 0.25, *p* = 0.011). Family CGs with lower health literacy experienced poor health (both mental and physical), higher anxiety, and greater fatigue, all with small effect sizes.

## Discussion

Approximately one-third of CGs of patients starting radiation treatment had limited to marginal health literacy, indicating they may struggle to read patient education materials and might require assistance. Before radiation treatment, patients and CGs receive extensive information, including details about the cancer diagnosis, diagnostic tests, complex treatment options, and potential side effects [30]. Despite the National Institutes of Health (NIH) recommending that healthcare materials be written at an eighth-grade readability level, most patient education materials are written at an 11.2 to 13.8-grade level [31]. Furthermore, most radiation treatment education materials used in cancer centers do not meet health literacy best practices, contributing to health disparities [32]. Consequently, CGs feel overwhelmed, experience information overload, and face uncertainty and unpreparedness [30]. During this stressful time period, low health literacy in CGs may result in poor understanding of the information provided, ultimately leading to poor outcomes for both patients and CGs [33].

The findings of this study highlight the elevated levels of anxiety experienced by CGs prior to the initiation of patient treatment (*M* = 53.48), which was higher than the mean score of 50 for the general US population. Our results align with a previous study that identified anxiety as a prevalent problem among cancer CGs prior to the start of cancer treatment, affecting more than 40% of them [34]. The impacts of education can be limited when CGs are anxious, as anxiety can impede their concentration and understanding of the new information provided [35]. Therefore, healthcare providers need to be aware of and manage CG’s anxiety before initiating education.

Our study identified a significant relationship between CGs’ health literacy, socioeconomic status, health, anxiety, and fatigue. These findings align with the Health Literacy Skills Conceptual Framework, which guided this study. According to the framework, background factors, including individual resources such as socioeconomic status, influence health literacy skills, which in turn impact health-related outcomes, measured here as mental and physical health, anxiety, and fatigue. These findings are also consistent with existing research that links lower health literacy and socioeconomic status to poorer health outcomes, increased anxiety, and fatigue in general populations and patients with cancer [36, 37]. This suggests that the challenges faced by CGs may be exacerbated by their health literacy and socioeconomic status, similar to patterns observed in the general population. Our findings suggest that CGs with lower health literacy and socioeconomic status may be particularly vulnerable during this period, as they may struggle more with the complex information and logistical challenges associated with preparing a patient for radiation treatment. This highlights the critical need for resources and interventions that can empower these CGs, ensuring they are equipped to fulfill their essential roles effectively. For example, interventions could include implementing health literacy screening tools during initial consultations to identify CGs who may need additional support. Tailored education programs, such as workshops or online modules, can provide practical guidance on caregiving tasks, treatment processes, and symptom management. Additionally, CG could benefit from simplified and visually engaging materials to enhance their understanding of complex healthcare information. Furthermore, integrating CG navigators or peer support groups can provide ongoing assistance, particularly for those with lower health literacy and socioeconomic challenges.

Our correlation analysis indicated significant positive relationships between both education and health literacy, and socioeconomic status and health literacy. However, in our structural equation model, the direct path from education to health literacy was not significant, while the path from socioeconomic status to health literacy remained significant. One possible explanation for this discrepancy is that the effect of education on health literacy is mediated by socioeconomic status. Higher education levels are likely to lead to higher socioeconomic status, which in turn improves literacy [38]. In addition, measurement error in the education variable might also contribute to the non-significant direct path. In our study, education was measured using broad categories: “less than high school,” “high school diploma/GED,” “college,” and “post-graduate.” While these categories provide a general sense of educational attainment, they also introduce some limitations, such as loss of precision, as they do not capture the finer differences within each educational level. The structural equation model accounts for measurement error, potentially leading to more accurate but non-significant estimates. However, our model specification and fit indices indicate a well-fitting model, suggesting that the lack of significance is not due to poor model fit but rather than the underlying relationships between the variables. Future research should consider using more granular categories or treating education as a continuous variable (e.g., years of education) to capture more detailed variations in educational attainment and provide more accurate estimates of its relationship with health literacy.

Surprisingly, previous caregiving experience and professional caregiver experience were not significantly associated with CG health literacy, despite the conceptual framework suggesting that prior knowledge influences health literacy. This finding suggests that familiarity with caregiving tasks, whether through personal or professional experience, does not necessarily translate into higher health literacy levels. It suggests that health literacy is not automatically enhanced by caregiving experience alone but may require targeted education and support to improve CGs'ability to manage health-related tasks effectively. It may be that oncology, or radiation oncology in particular, comes with its own unique language and challenges for health literacy. Healthcare providers should provide tailored support and education to all CGs, recognizing that experience does not automatically equate to proficiency in health literacy in this unique context.

The study findings emphasize that it is crucial to acknowledge CG health literacy during patient healthcare visits. According to a study that utilized a national survey, the majority of cancer caregivers (87.6%) were actively involved in cancer treatment decision-making [39]. The high level of involvement underscores the necessity of assessing and addressing CG health literacy to ensure that they can fully comprehend the complexities of cancer treatment options, potential outcomes, and care instructions. In addition, healthcare providers should adhere to the AHRQ Health Literacy Universal Precautions Toolkit [40] for all CGs. Employing plain language and the teach-back method, providing written materials at appropriate readability levels, and offering additional support and resources are crucial for improving comprehension among CGs with low health literacy. Furthermore, healthcare providers should be mindful of information overload during patient and CG education at the start of treatment, as a heavy volume of information is often delivered within a short period [10, 13, 41]. This information overload can further increase stress and misunderstanding of information [41]. To minimize information overload, strategies such as providing tailored education, offering written handouts with pictographic materials, and limiting the amount of information given at one time can be used [41].

### Study limitations

This study has several limitations. First, the study findings may have limited generalizability to the broader cancer CG population due to the use of a convenience sampling method. The demographic characteristics of the study sample overrepresent females, white individuals, and non-Hispanic/Latino CGs, which may limit the generalizability of the findings to more diverse CG populations. Additionally, more than half of the participants had a college degree or higher and an annual household income exceeding $50,000. Second, the study did not assess CGs’ primary language, although language proficiency is a key factor affecting health literacy and the ability to navigate complex healthcare information. Third, the study used self-report measures which may be subject to response bias. Lastly, the study design was cross-sectional, so future research could benefit from a longitudinal approach to better understand the comprehensive impact of health literacy on cancer CGs and patients over time. While the study has certain limitations, the study employed validated tools to ensure the reliability and validity of the measurements. Also, we utilized structural equation modeling, a robust analytical method that allows for the examination of complex relationships between variables. These strengths enhance the study’s rigor and provide valuable insights into the relationships between CG’s health literacy, demographic variables, and health-related outcomes.

## Conclusion

In summary, our study highlighted the critical issue of limited or marginal health literacy among one-third of cancer CGs prior to patients’ radiation treatment. This finding is particularly concerning given the significant relationships we identified between CGs’ health literacy and key factors such as socioeconomic status, overall health, anxiety, and fatigue. These results suggest that CGs with lower health literacy may be at greater risk for poor health outcomes, higher levels of anxiety, and increased fatigue, which could ultimately impact their ability to provide effective care for the patients. Given the interconnected nature of these factors, it is essential to develop targeted interventions that address health literacy while also considering the broader socioeconomic and psychological context of CGs. Systemic changes could also involve improving the readability of healthcare materials by presenting them in plain language, incorporating visual aids, and tailoring contents to diverse cultural contexts. Additionally, integrating CG support into healthcare policies could include providing CG training programs and routinely assessing CG needs. Future research should further explore these relationships through longitudinal studies to better understand the long-term impact of health literacy on CG and patient outcomes. Incorporating additional predictors, such as cultural and linguistic factors (e.g., language proficiency and cultural beliefs) and healthcare system factors (e.g., communication with healthcare providers and navigation of healthcare systems), could enhance the final model by providing a more comprehensive understanding of the factors influencing health literacy. Additionally, there is a need for practical strategies that healthcare providers can use to identify and support CGs with limited health literacy, ensuring they have adequate resources and knowledge to navigate complex cancer trajectories.

## Data Availability

No datasets were generated or analysed during the current study.
